# EMG-based simultaneous and proportional estimation of wrist/hand kinematics in uni-lateral trans-radial amputees

**DOI:** 10.1186/1743-0003-9-42

**Published:** 2012-06-28

**Authors:** Ning Jiang, Johnny LG Vest-Nielsen, Silvia Muceli, Dario Farina

**Affiliations:** 1 Strategic Technology Management, OttoBock HealthCare GmbH, Max-Näder Str. 15, Duderstadt, 37115, Germany; 2 Center for Sensory-Motor Interaction (SMI), Dept. of Health Science and Technology (HST), Fredrik Bajers Vej 7 D3, Aalborg University, Aalborg, 9220, Denmark; 3 Department of Neurorehabilitation Engineering, Bernstein Center for Computational Neuroscience, University Medical Center Göttingen, Georg-August University, , Von-Sieblod-str. 4, Göttingen, 37075, Germany

## Abstract

We propose a method for estimating wrist kinematics during dynamic wrist contractions from multi-channel surface electromyography (EMG). The algorithm extracts features from the surface EMG and uses dedicated multi-layer perceptron networks to estimate individual joint angles of the 3 degrees of freedom (DoFs) of the wrist. The method was designed with the aim of proportional and simultaneous control of multiple DoFs of active prostheses by unilateral amputees. Therefore, the proposed approach was tested in both unilateral transradial amputees and in intact-limbed control subjects. It was shown that the joint angles at the 3 DoFs of amputees can be estimated from surface EMG recordings , during mirrored bi-lateral contractions that simultaneously and proportionally articulated the 3 DoFs. The estimation accuracies of amputee subjects with long stumps were 62.5% ± 8.50% across all 3 DoFs, while accuracies of the intact-limbed control subjects were 72.0% ± 8.29%. The estimation results from intact-limbed subjects were consistent with earlier studies. The results from the current study demonstrated the feasibility of the proposed myoelectric control approach to provide a more intuitive myoelectric control strategy for unilateral transradial amputees.

## Introduction

The Electromyographic signal (EMG) has long been used to control powered prostheses, particularly those for upper-extremities, because it contains information on the neural control of movement [1]. This control scheme is often referred to as myoelectric control [2]. In the past 30 years, it has been demonstrated that pattern classification of EMG signals can consistently achieve very high classification accuracy [3]. For example, an accuracy greater than 95% can be achieved when classifying 6 hand/wrist contractions of intact-limbed subjects from EMG signals [4,5]. The performance of these approaches on amputees is also promising. Ajiboye and Weir reported classification accuracy ranging from 74% to 99%, for two amputee subjects performing 5 wrist functions [6]. Employing a mirrored training paradigm and artificial neural network (ANN), Sebelius *et al.*[7] reported that both traumatic and congenital amputees can achieve > 80% accuracy on selected 4-6 wrist/hand movements. More recently, Li *et al.*[8] reported > 88% accuracy in 5 transradial amputees over 6 wrist/hand motions.

Despite the promising performance of these myoelectric control algorithms, currently no commercial myoelectric prosthesis is based on EMG pattern classification. Rather, commercial devices are still based on the conventional, very simple direct control approach (based solely on EMG amplitude), which has been used for more than half century [2]. There are many reasons for this contrast between the industrial and academia state-of-the-art.

Although extensive research activities aimed at improving applicability of pattern classification based myoelectric control, such as feature stability [9], training adaptation [10,11], and false positive (activation) control [12], the pattern classification paradigm is drastically different from the way in which the neuromuscular system controls the muscles. A pattern classification paradigm indeed provides a sequential and on/off control of a predefined set of muscle activation patterns (motions). In contrast, the neuromuscular system smoothly articulates (proportional control) multiple degree-of-freedoms (DoFs) simultaneously (simultaneous control). As such, the prosthesis users often feel that the provided control is not intuitive, resulting in long, some times discouraging training/learning periods.

To realize a bio-mimic and more intuitive myoelectric control paradigm, the control algorithms should provide simultaneous and proportional control of multiple DoFs, as it has been recently addressed in a few studies. Based on the concept of muscle synergy [13], Jiang *et al.*[14] demonstrated that a modified non-negative matrix factorization algorithm can simultaneously estimate the torque produced at the three DoFs of the wrist in intact-limbed subjects. Nielsen *et al.*[15] extended the method proposed by Jiang *et al.*[14]. In that study, a bilateral, mirror-training strategy was employed, and it was shown the force from contra-lateral limb can be estimated reliably using the EMG from the ispi-lateral limb, during mirror movements. This is fundamental for the application in unilateral amputees where the training of the algorithm obviously can not be performed between EMG and force recorded from the phantom limb. Further, Muceli *et al.*[16,17] demonstrated in intact-limbed subjects that mirror movements can be used for training simultaneous and proportional control in dynamic tasks to estimate joint kinematics instead of force.

The current study aims at developing a method for proportional and simultaneous control of three DoFs of the wrist joint (flexion/extension, radial/ulnar deviation, and supination/extension), and at demonstrating its clinical applicability in unilateral amputees. For this purpose, both transradial amputee subjects (with different levels of amputations), and intact-limbed control subjects were tested using an experimental protocol where multi-channel surface EMG was used to estimate the joint kinematics (joint angles) during mirrored bilateral, simultaneous articulations of the wrist.

## Methods

### Description of participating subjects

Six individuals (3 male, 3 female; age range: 31-52 years; referenced A1 - A6) with unilateral transradial amputation (five traumatic, and one congenital malformation (A3)) participated in the experiment. This type of limb deficiency was considered because it represents a large portion of the upper-limb amputations. The 6 amputee subjects were further grouped into two groups: short stump group (SS), including subjects A1, A2, and A3; long stump group (LS), including subjects A4, A5 and A6. Although the stump of the congenital subject (A3) was long, the musculature of her right forearm was substantially smaller than the subjects in the LS group. Therefore, she was assigned to the SS group. All amputee subjects are users of conventional myoelectric prosthesis. The information of the amputee subjects are summarized in Table 1.

**Table 1:** Amputee subjects data

**SUBJECT ID**	**AMP. TYPE**	**TIME OF AMP.**	**POSITION OF AMP.**	**GROUP**
A1	Traumatic	N/A	5cm distal from elbow	SS
A2	Traumatic	2003	ca .10cm distal from elbow	SS
A3	Congenital	Congenital	forearm ≈20cm	SS
A4	Traumatic	2007	2/3 distal from elbow	LS
A5	Traumatic	2002	ca. 20cm distal from elbow	LS
A6	Traumatic	2003	ca. 20cm distal from elbow	LS

In addition to the 6 amputee subjects, 5 able-bodied subjects (2 male, 3 female; age range: 24-40 years; all right-handed, H1 - H5), with no known neuromuscular disorders, also took part in the experiments, as control subjects. This subject group was denoted control group (CG). All 11 subjects signed an informed consent form prior to participating in the experiments. The experimental protocol was approved by the local ethics committee.

### Experimental setup

The study involved the concurrent recording of multichannel surface EMG signals of upper limbs and kinematics of unrestrained and dynamic, non-loaded contractions of hand/wrist during mirrored bilateral wrist movements.

#### EMG recordings

For all subjects, 7 pairs of Ag-AgCl surface bipolar electrodes (Type: Ambu NeuroLine 720) were placed on each forearm. At the intact side, the electrode pairs were placed around the thickest part of the forearm (approximately 1/3 distally from the elbow), equi-spaced in a circle around the forearm, similarly to [11,14]. Equi-spaced electrode placement was used, rather than targeting at specific muscles, because 7 electrode pairs provided necessary coverage of the area of interest, as shown in previous studies [14,15]. The first pair was placed 1 cm medially from the ulnar bone (found by palpation) and the remaining six pairs were positioned sequentially in the pronation direction. On the amputated side, the electrodes were placed on the same place as at the intact side, whenever possible. When the stump was too short, the electrodes were placed around the region where most musculature existed (found by palpation). A reference armband (placed on one of the wrists for the intact-limbed subjects and on the wrist of the intact side of the amputees) was used for common reference point. All electrodes were connected via high noise-rejection cables to an EMG-amplifier (EMG-USB, 128 channel, OT Bioelettronica), where the EMG signals were sampled at 2048 Hz, amplified at 2*k*, and digitized with 12-bit precision.

#### Kinematics recordings

**Figure 1:**
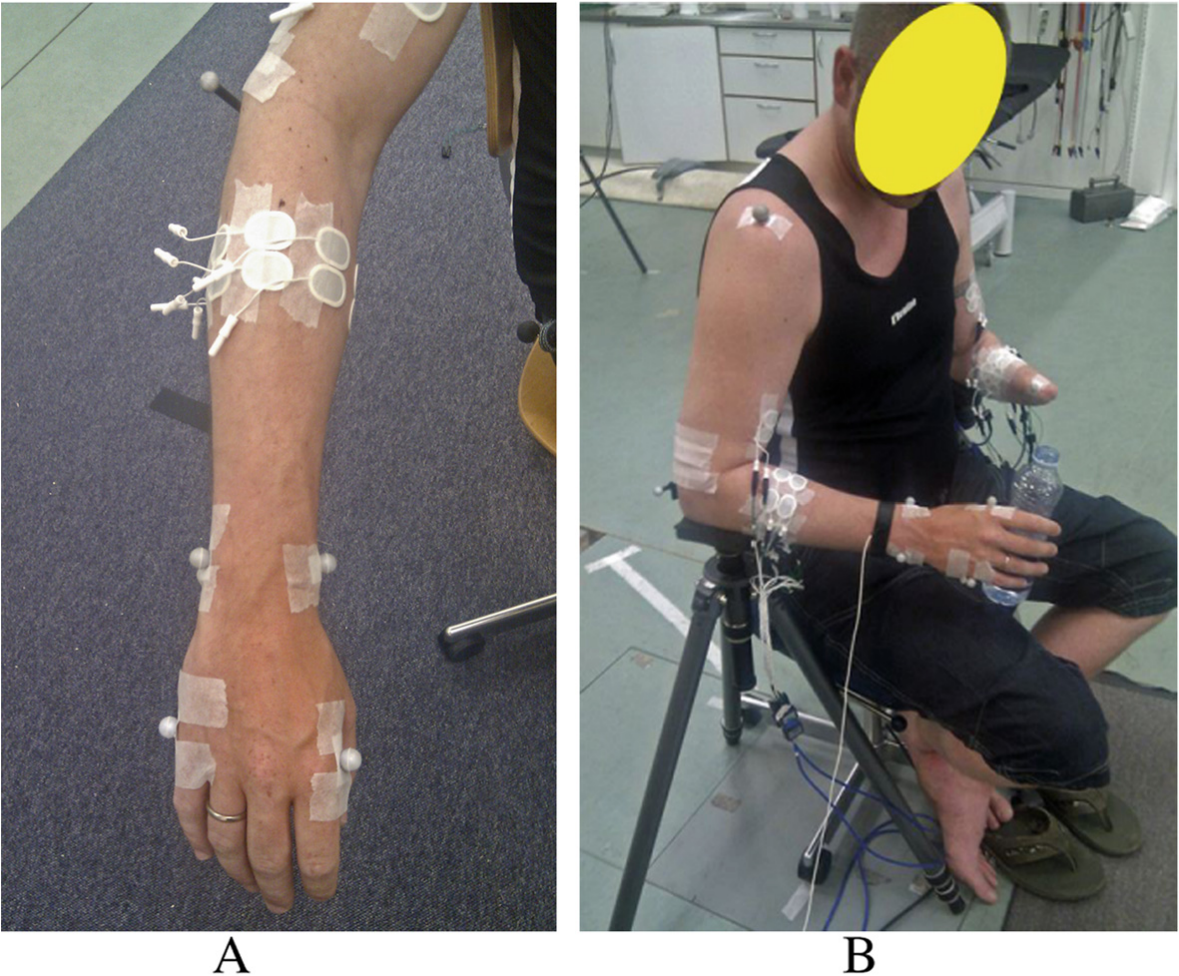
**Position of markers and electrodes.** (**A**) the markers and electrodes on the intact limb of an amputee subject. Same setup for both limbs of the able-bodied subjects. (**B**) the markers and electrodes for an amputee subject: subject A3.

A 8-camera motion capture system (Qualisys AB, Sweden) was used to record kinematics of the limbs during the movements. Passive-reflective spherical markers (diameter 12 mm) were placed on both arms of the subjects. For the intact side of the amputee subjects, 7 markers were placed on the following anatomical skeletal landmarks (found by palpation): one on the shoulder (prominent point of the Scapular Acromion); two parallel to the elbow (prominent points of the medial and lateral Epicondyle of Humerus, denoted by MEP and LEP); two at the wrist (distal Styloid processes of Ulna and Radius, denoted by STU and STR); and two at the hand (distal laterally and medially prominent points of the second and fifth Metacarpal bone, denoted by RMC and UMC). On the amputated side, the first 5 markers were placed at the same place as the intact side, and the two additional markers were placed on the distal end of the stump, over the prominent points of the Ulna and Radius bones (found by palpation). For able-bodied subjects, 7 markers were placed on each arm, at the same places as the intact limb of the amputee subjects. The position of the markers and electrodes is shown in Figure 1. The marker trajectories were digitized in a 3D coordinate space and sampled at 256 Hz by a 12-bit A/D converter. An external synchronization signal (20 Hz square wave, ± 5V) was provided to both the EMG acquisition system and the motion capture system so that the EMG traces and the kinematics could be synchronized.

### Experimental protocol

During an experimental session the subject sat in a standard chair, placed on a podium 0.3 m above floor level, with the elbows resting on two armrests, as shown in Figure 1. The armrests were adjusted so that the subject felt relaxed, and his/her shoulders and upper arms were in symmetric positions. The eight cameras of the motion capture system were mounted on tripods, at various heights (0.5 - 2.5 m) above the floor, and placed in a circular pattern (average radius 2.5 m) around the subject. The exact positions and heights of the cameras were optimized during preliminary tests to provide the maximum coverage of all markers during the intended movements.

During a recording session, the subjects performed a series of mirrored bilateral dynamic wrist contractions. The contractions involved articulations of the 3 DoFs of the wrist: flexion/extension (DoF1), radial/ulnar deviation (DoF2) and wrist pronation/supination (DoF3). The DoFs were either articulated separately, or simultaneously. The amputee subjects were instructed to imagine moving their phantom limbs in a mirrored fashion along with their intact side. The able-bodied subjects were instructed to do mirror movements. At the beginning of an experimental session, the subjects familiarized themselves with the protocol by performing the mirrored bilateral contractions. At the same time, the cameras’ positions were optimized. Then, the subject was instructed to perform three contraction groups, containing activations of both single and combined DoF(s). A detailed description of the contractions is reported in Table 2. The subject was instructed to perform these tasks at low to medium speed (the speed was subjectively controlled by the subject). The spatial marker trajectories were visually inspected by the attending experimenter after each contraction, and was repeated if deemed unsatisfactory due to excessive gaps in the acquired marker trajectories. Each trial lasted approximately 65 s and was separated to the next by resting periods of 2-3 min to avoid fatigue. A total of 10 trials were performed with the elbows flexed at 90° and the arms 10° abducted from the torso.

**Table 2:** List of the contractions performed

**GROUP**	**DESCRIPTION**	**ACTIVE DoF**
1.	Sinusoidal contractions	1: flexion/extension (DoF1)
	along a single DoF	2: radial/ulnar deviation (DoF2)
	(freq. ≈ 0.5 − 1 Hz)	3: pronation/supination (DoF3)
2.	Combined activation	4: DoF1 + DoF2
	of two DoFs, in which	5: DoF2 + DoF1
	one DoF was articulated	6: DoF1 + DoF3
	sinousoidally, and the other	7: DoF3 + DoF1
	was fixed at positions close	8: DoF2 + DoF3
	to maximal range of motion	9: DoF3 + DoF2
3.	Cyclic contractions of	10: DoF1 + DoF2 + DoF3
	unconstrained dynamic	
	wrist movements.	
	(freq. ≈ 0.5 − 1 Hz)	

### Data processing

#### EMG features

The EMG signal was band pass filtered (10 - 450 Hz, second order Butterworth filter). To estimate the kinematics at the wrist joint, the time domain (TD) feature set [18], and the 6 autoregressive coefficients (AR), namely the TDAR feature set, were used [15]. The analysis window was 100 ms long, with 60 ms overlap. In preliminary analyses (not reported) of this study, more complex features, such as wavelet marginals or coefficients, did not provide significantly better estimation performance. Therefore, only the TDAR feature set was used, both for the amputee subjects and for the able-bodied subjects.

#### Kinematic data processing

**Figure 2:**
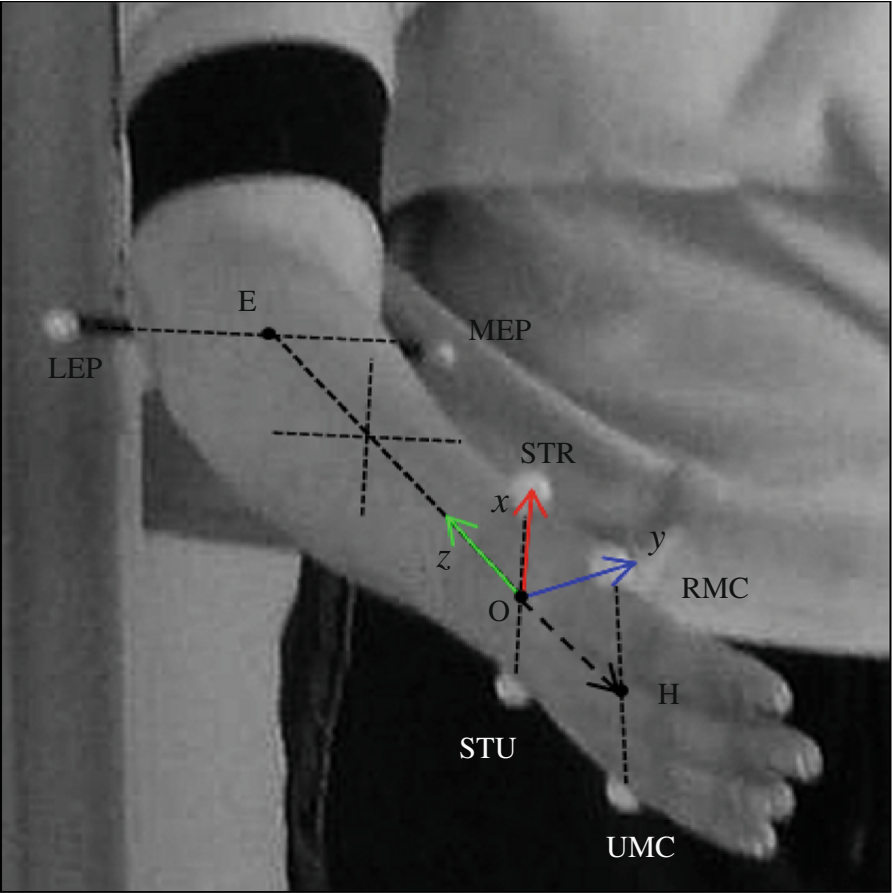
**The coordinate system for joint angle calculation.** MEP and LEP: medial and lateral Epicondyle of Humerus. STR and STU: distal Styloid processes of Radius and Ulna. RMC and UMC: the distal laterally and medially prominent points of the second and fifth Metacarpal bone. The mid-point of MEP and LEP is marked as E (elbow). The mid-point of STR and STU is marked as O (origin). The mid-point of RMC and UMC is marked as H (hand). The *z*-axis point from O to E, the *y*-axis is orthogonal to *z*-axis and the line defined by STR and STU, and *x*-axis is orthogonal to *z*-axis and *y*-axis.

The kinematic angular displacement for each of the three DoF’s were calculated from a coordinate system, illustrated in Figure 2. The Origin of the system is at the center of the wrist, midway between the STR and STU, denoted by *O*; the *z*-axis set as the center axis of the forearm, positive in the proximal direction, pointing from *O* to the *E*, the midway between MEP and LEP; the *y*-axis set as the dorsopalmar axis, positive in the anterior direction; and the *x*-axis set as the mediolateral axis of the wrist, positive in the lateral direction. Denoting the mid-point between RMC and UMC by *H*, the angle of DoF1, *α*_1_, is defined in (1), where *H*_*y*_ and *H*_*z*_ is the projection of *H* on *y*-axis and *z*-axis, respectively. It is assumed that this angle can not be larger than 90° at either side. Ideally, a positive angle indicates flexion, and a negative angle indicates extension. The angle of DoF2, *α*_2_, is defined in (2), where *H*_*x*_ is the projection of *H* on *x*-axis. It is assumed that this angle can not be larger than 90° at 90° at either side. Ideally, a positive angle indicates radial deviation, and a negative angle Denoting the vector from MEP to LEP as $\vec{w}$, and the vector from STU to STR as $\vec{l}$, the angle of DoF3, *α*_3_, is defined as angle between the two vector $\vec{w}$ and $\vec{l}$, as in (3). Ideally, an angle smaller than 90° indicates supination, and an angle greater than 90° indicates pronation.

(1) $$\begin{array}{lcr} \alpha_{1} & = & \text{atan}(\frac{H_{y}}{H_{z}}) \end{array}$$

(2) $$\begin{array}{lcr} \alpha_{2} & = & \text{atan}(\frac{H_{x}}{H_{z}}) \end{array}$$

(3) $$\begin{array}{lcr} \alpha_{3} & = & ∠\left(\vec{w},\vec{l}\right) \end{array}$$

The kinematic data were offline low-pass filtered (6 Hz, 2nd order Butterworth filter [19]). All EMG and kinematic data were re-sampled to 1024 Hz, and synchronized through the common synchronization signal.

#### Multi-layer perceptron network training

Multi-layer perceptron (MLP) artificial neural networks were used to learn the association between the EMG features and the kinematic signals. In this study, a ’dedicated MLP approach’ was taken in estimating the simultaneous articulations of the 3 DoFs, similar to the approach described in [16]. It was shown that this approach is superior than the ’one MLP for all DoFs’ approach in [14,15].

To investigate the possibility of using EMG from one arm to estimate the arm kinematics of the other arm (as is necessary for unilateral amputees), two MLP training scenarios were performed: contra-lateral training and ipsi-lateral training, as it was done for intact-limbed subjects in [15,16]. For contra-lateral training, the MLPs were trained using EMG features from the amputated side (for amputee subjects), or the dominant side (intact limbed subjects). For ipsi-lateral training, the MLPs were trained using EMG features obtained from the intact side (for amputee subjects), or from the non-dominant side (for intact limbed subjects).

In addition, to investigate the ability of the MLP to estimate the kinematics at different DoFs, the MLPs were trained and tested on various data sets where the DoFs were activated selectively and/or simultaneously. This determined 4 analysis scenarios based on the DoF(s) considered. For example, the analysis scenario was identified as *DoF12* when only contractions involving DoF1 and DoF2 were used for analysis (contractions 1, 2, 4, and 5; estimating for the first two DoFs). Similarly, the analysis scenario *DoF13* consisted of the analysis of the contractions (numbered as 1, 3, 6, and 7), where only DoF1 and DoF3 were activated. When all 10 contraction types were used in the analysis, the scenario is indicated as *DoF123*, and corresponded to the estimation of all DoFs simultaneously.

**Figure 3:**
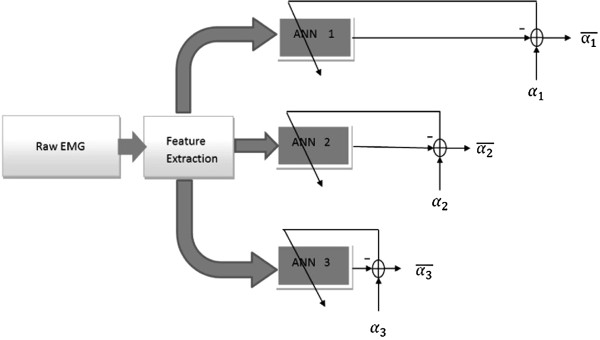
**The training of the MLPs.** The surface EMG features were the inputs to the MLPs, which used one of the joint angles as training target. The joint angles were obtained from the intact side (amputee subjects), or non-dominant side (intact-limbed subjects). When the EMG from the same side as kinematics was used for training, it was called ipsi-lateral training. When the EMG from the opposite side was used for training, it was called contra-lateral training. *α*_1_, *α*_2_, and *α*_3_ are the joint angles of the three DoFs, as defined Eq. (1), Eq. (2), and Eq. (3), respectively. $\bar{\alpha_{i}}$ are the respectively estimates.

For each combination of contra-lateral vs. ipsi-lateral and DoF-wise training scenarios described above of each subject, a data set (EMG feature and joint angles) was obtained. The EMG features were the inputs to the MLP, and the corresponding joint angle was the estimating target. The data set was divided in five blocks for a five-fold cross-validation procedure, with one of the five blocks as testing set for the MLPs, and the remaining four blocks as the corresponding training and validation set. For each fold, the training and testing was repeated 30 times with different initial weights, resulting in 30 MLPs with different internal parameters for each DoF. The MLPs producing the highest $R_{i}^{2}$ (the estimation performance metric discussed below), were kept as the ’winners’ of the current fold. The global performance index, *R*^2^, of the current fold were obtained from the estimations by these winning MLPs. Thus, for each subject and each combination of training scenarios, 5 *R*^2^ values were retained. The training and testing of the MLP are schematically represented in Figure 3. In the preliminary analysis (not shown), it was determined that 3 neurons in the hidden layer of the MLP was a good balance between the performance and computational efficiency. Moreover, 100 ms was chosen as the processing window for the EMG feature extraction, as it is has been suggested as an acceptable delay for multi-function prosthetic application [20].

#### Performance index

The multivariate *R*^2^ index, originally proposed by d’Avella *et al.*[21], and applied in [14,15], was used to quantify the estimation performance of the MLP. This *R*^2^ index has previously been shown to be an effective performance measure, as it represents the percentage of total variation of the targets captured by the estimation. It is thus a global indicator of the quality of the estimation. The global *R*^2^ is defined as follows:

(4) $$R^{2}=1-\frac{\overset{D}{\underset{i=1}{\sum }}\overset{N}{\underset{t=0}{\sum }}{\left(\hat{\alpha_{i}(t)}-\alpha_{i}(t)\right)}^{2}}{\overset{D}{\underset{i=1}{\sum }}\overset{N}{\underset{t=0}{\sum }}{\left(\alpha_{i}(t)-\bar{\alpha_{i}(t)}\right)}^{2}}$$

where *D* is the number of targets (same as the number of DoFs), *N* is the number of data samples, *α*_*i*_(*t*) is the joint angle of the *i*th DoF, $\hat{\alpha_{i}(t)}$ is the corresponding estimate by the MLP, and $\bar{\alpha_{i}(t)}$ is the temporal average of *α*_*i*_(*t*). The numerator in the second term of the right hand side of Eq. (4) is the total *mean square error* (MSE) of the estimates and the denominator is the total variance of the targets. Similarly, the $R_{i}^{2}$ for individual DoF is defined as [16,17]:

(5) $$R_{i}^{2}=1-\frac{\overset{N}{\underset{t=0}{\sum }}{\left(\hat{\alpha_{i}(t)}-\alpha_{i}(t)\right)}^{2}}{\overset{N}{\underset{t=0}{\sum }}{\left(\alpha_{i}(t)-\bar{\alpha_{i}(t)}\right)}^{2}}$$

The reason for using *R*^2^ as the performance metric over mean square error (MSE), is that MSE produces a biased result when the target values are very small [21]. In the current study, both the DoF-wise performance in (5) and the global performance in (4) are reported.

### Statistical analysis

The main hypothesis is that the performance in the contra-lateral training scenario in amputees is comparable to that of intact-limbed subjects. Separate ANOVA tests were conducted for each of the 4 DoF-wise scenarios, namely, *DoF*12, *DoF*13, *DoF*23, and *DoF*123. In these tests, the response variables were *R*^2^, and the two factors were Contra-/Ipsi- Lateral and Subject Group (SS/LS/CG). The subjects were nested with the Subject Group factor, and were repeated measures within Contra/Ipsi-lateral factor. The significance level was set at 95%. As shown in the Results Section below, for all the tests ran, there was always a strong and significant interaction between the two factors, in which case it is not advisable to interpret the main effects directly. Therefore, the effect of Contra-/Ipsi-lateral was analyzed by fixing the Subject Group factor, and the effect of Subject Group factor was analyzed by fixing the Contra-/Ipsi-lateral factor, with one-way ANOVA analyses.

## Results

**Figure 4:**
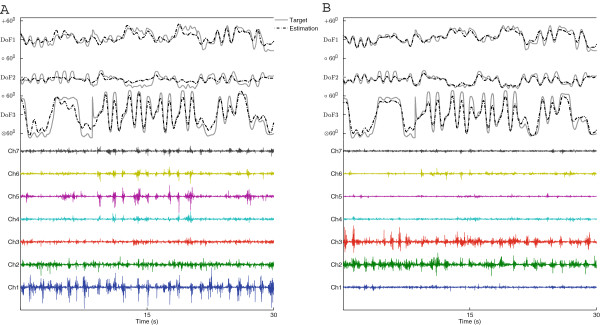
**Representative experimental data.** (**A**) the contra-lateral case. The 3 measured joint angles of the right arm (intact side) of subject A6 are reported as gray solid lines, and the corresponding estimated angles are indicated against the measured ones, in black dashed lines. Below are the 7-channel raw EMG from the subject’s left arm (amputated side), from which the estimated angles of the intact side were obtained. (**B**) the ipsi-lateral case. The same measured joint angles as the ones in panel **A**. The estimated joint angles in black dashed lines were obtained by the EMG from the right arm (intact side). The signals shown are from one portion (contraction 10) of the testing set from one fold of the five-fold cross-validation.

**Figure 5:**
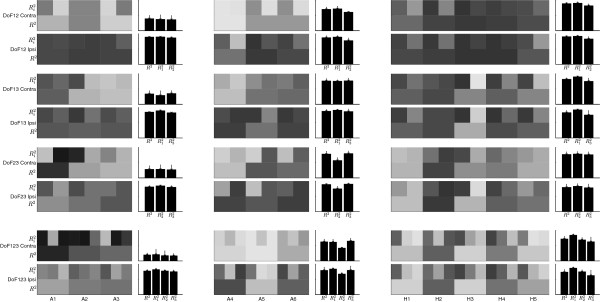
**Summary of the results.** The top six rows are the scenario when only 2 of the 3 DoFs are considered, *i.e.DoF*12, *DoF*13, and *DoF*23. The bottom two row are the scenario when all 3 DoFs are considered (*DoF*123). Each column is the result of one subject. The subjects are grouped in SS, LS and CG, from left to right. The summary for each group is presented in the bar plots next to the results of the individual subjects of each group. Black and white in the gray scale represents 0 and 1 respectively. The group standard deviation is represented by the vertical lines over each bar. The vertical ranges of all the group average plots are from 0 to 100%.

Examples of the EMG recorded, the corresponding wrist angles, and the corresponding estimation, are presented in Figure 4 for one amputee subject in the LS group (A6). The results of all subjects are presented in Figure 5.

The *R*^2^ value for *DoF*123 of the three subject groups were: SS 19.7% ± 5.76% (77.5% ± 4.21%), LS 62.5% ± 8.50% (79.3% ± 10.4%) and CG 72.0% ± 8.29% (73.6% ± 8.59%), for Contra-lateral (Ipsi-lateral) scenarios, respectively. All results are summarized in Figure 5. It is worth noting that for almost all training scenarios from a particular subject, the coefficient of variation of the *R*^2^ within any 5-fold cross validation was usually smaller than 10%. The only exceptions were for the SS Subject Group in Contra-Lateral scenario, where the performance were poor. This small variation within the cross-validation indicated a rather homogeneous data sets.

As discussed above, all the two-way ANOVAs revealed significant interactions between the two main factors. Therefore, separate one-way ANOVAs were performed for each factor, by fixing the level of the other factor, with an increased significance level at 97.5% (Bonferroni correction).

### Subject group effect

**Figure 6:**
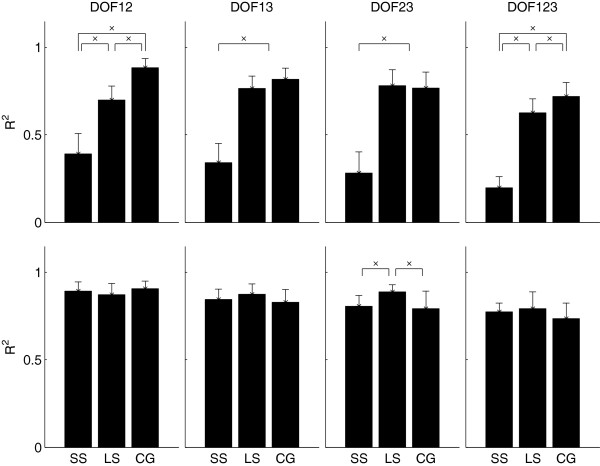
**The effect of the Subject Group when the Lateralization factor was fixed.** Top is Contra-lateral, and the bottom row is Ipsi-lateral.

The Subject Group effect with fixed levels of Contra-/Ipsi- Lateral factor is reported in Figure 6. When the Contra-/Ipsi- Lateral factor was fixed at contra-lateral, regardless of the DoFs considered, the performance across the Subject Groups was significantly different (*p* < 10^−3^). Tukey’s post-hoc comparison showed that the CG and LS groups performed significantly better than SS regardless the DoFs considered. The LS group performed comparable to CG, but statistically significant differences existed in the case of *DoF123*, and *DoF12*. However, there were no significant differences between the LS and CG group in the cases of *DoF13* and *DoF23*. These results indicated that the performance of the SS group was always inferior than the other two groups, while in some cases, the performance of the LS and CG were not significantly different.

When the Contra-/Ipsi- Lateral factor was fixed at ipsi-lateral, the performance across the groups was not statistically significant for *DoF123* (p = 0.10), *DoF12* (p = 0.15), and *DoF13* (p = 0.12). But there was significant difference for *DoF23* (p = 0.002). Post-hoc analysis for this case showed that the LS group performed significantly better than the CG and SS groups, while no significant difference existed between the CG and SS groups. These results indicated that, in most cases, there was no difference across the subject groups when ipsi-lateral EMG was used to estimate the joint angles, as it was expected (the control condition).

### Contra-/Ipsi lateral effect

**Figure 7:**
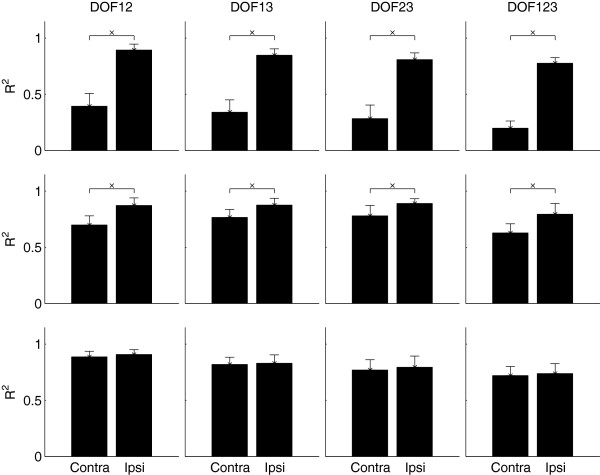
**The effect of the Contr-/Ipsi-lateral factor when the Subject Group factor is fixed.** The 3 rows, from top to bottom, represent the 3 Subjects Groups: SS, LS, CG, respectively.

For both SS and LS group, there was always a significant difference between contra-lateral and ipsi-lateral estimates (*p* < 10^−3^). For the CG group, however, no significant difference could be found in all cases. The *p*-values were 0.52, 0.12, 0.58, and 0.36 for *DoF12*, *DoF13*, *DoF23*, and *DoF123*, respectively. The Lateralization effect is summarized in Figure 7.

## Discussion

This paper is the first that demonstrates the feasibility of estimating wrist/hand kinematics of transradial amputees using surface EMG during mirrored bi-lateral movements with simultaneous and proportional activations of the 3 wrist DoFs. Previous research by our group [15-17] showed that this is feasible in case of intact-limbed subjects. When a simplified version of the proposed training strategy (limited to two DoFs and to isometric contractions, with one MLP for all DoFs) was applied to one subject with congenital malformation of the forearm, performance worsened, but was still encouraging [15]. The present paper provides a systematic validation of mirror training for estimating wrist kinematics in amputee subjects. The proposed strategy was tested in individuals with different levels of amputation.

The results on intact-limbed subjects in the current study are consistent with previous studies. For the CG group and *DoF12*, the *R*^2^ values were 88.5% ± 4.28% (90.6% ± 3.68%) in contra-lateral (ipsi-lateral) cases. The corresponding values in [15] were 90% ± 2% and 93% ± 2%. The performance worsened in *DoF123* scenario, with *R*^2^ of 72.2% ± 8.29% (73.6% ± 8.59%) in contra-lateral (ipsi-lateral) cases. This result, which is also in agreement with previous work [16], is likely explained by the fact that the pronators and supinators are deep muscles whose activities can be masked by flexors and extensors which are superficial. No differences were found between contra-lateral and ipsi-lateral training scenarios for the intact-limbed subjects.

For amputees subjects, we investigated performance both for the intact and amputated sides, in order to allow a direct comparison with able bodied subjects. As expected, amputees performed better with their intact limbs. As shown in Figure 6, no significant differences were found for the performance among intact-limbed subjects and the intact side of amputees, with the only exception for the scenario *DoF23* where LS amputees performed better than the other two groups.

For amputees with long stumps (LS group), the contra-lateral estimation accuracy (*R*^2^) was 62.5% ± 8.50% for *DoF123*. This was lower than the CG group (72.2% ± 8.29%), which is explained by noting that the amputee subjects have not been using the remnant muscles on their amputated side for such complex motor tasks after the amputation. Among all the scenarios, the LS group showed the worst performance for tasks involving radial/ulnar deviation. The $R_{2}^{2}$ values of this DoF were: 60.6% ± 2.34% , 57.0% ± 9.52%, 41.3% ± 3.32% for *DoF12DoF23*, and *DoF123*, respectively. However, for the intact-limbed subjects, pronation/supination was consistently the worst estimated DoF, as also noted in previous studies [14,16]. Figure 5 confirmed that pronation/supination was better estimated than radial/ulnar deviation in the LS group. Whether this counter-intuitive result is inherent to limb deficiency or only due to small sample size needs further investigation.

The striking differences between the performances of the SS and the LS group in contra-lateral scenario highlights the myoelectric control paradox: higher amputation requires more functional restoration, while at the same time leaves less signal sites to acquire EMG signals. Subjects A1 and A2 had stumps approximately 5 and 10 cm long, i.e. they did not retain enough remnant muscles in the forearm from which neural control information could be extracted. While the subject with the congenital malformation (A3) has a longer stump, she reported difficulties in imagining the movements with the malformed limb since this was the first time the subject was asked to do such tasks.

Taken together, our results showed that as long as sufficient muscles remain in the stump of transradial amputees, surface EMG recorded from the stump can be used to extract simultaneous and proportional control information for all 3 DoFs of the wrist. In particular, the LS group performed similarly to the CG group when only DoF1 and DoF3 were considered, which are functionally more important than DoF2. It is possible they could achieve even better performance after repetitively using the proposed training paradigm. For individuals with shorter stumps, it is needed to increase the amount of information for control, e.g. by accessing the peripheral nervous system with intra-neural electrodes [22], or by targeted muscle reinnervation [23].

Most of the previous myolectric control strategies that have been tested in amputees rely on the pattern classification paradigm (to estimate a set of classes), so a quantitative comparison of performance is not feasible. Reported classification accuracies are usually high (> 90*%*). Although the performance reported in this study seems lower (but not comparable anyway given the different metrics), it is worth noting that we evaluated performance during dynamic contractions while classification accuracy is usually calculated on static contractions. Moreover, our control strategy provides simultaneous and proportional control of multiple DoFs and not sequential discrimination of a fixed number of tasks. In addition to higher functionality, this control approach is also more intuitive than those based on pattern classification in that it provides continuous and simultaneous control of functions. Currently, the performance was assessed through offline processing of pre-recorded signals. However, the proposed system is suitable for real-time application: the analysis window was limited to 100 ms, the features (TDAR) were simple, and only 7 channels were used.

To further validate the clinical applicability of the proposed approach, the next step is to establish a relationship between the algorithmic performance (e.g. *R*^2^) and functional performance, or usability, from which an acceptable *R*^2^ value for prosthetic control can be established. In pattern recognition based myoelectric control, it was shown that the algorithmic performance (classification accuracy), at the very best, has a very weak correlation with the usability [24]. This means that a system with 95% classification accuracy does not necessarily have a better usability than a system with 90% accuracy. Since the current approach allows intuitive, simultaneous and proportional control directly on the physiological DoFs, it is possible that a stronger relationship between the algorithmic performance and usability can be found. This issue will be the subject of further investigation.

For clinical feasible applications of the proposed approach, the DoF of hand open/close has to be included. This is because this DoF is the most important function for amputees. In a recent study [17], we have demonstrated that, for intact-limbed subjects, the kinematics of this DoF can be estimated with similar accuracies as the other DoFs.

Other ongoing work is underway to provide an online implementation of the proposed system to evaluate its real time performance on both amputees and intact-limbed subjects, and its clinical applicability.

## Competing interests

NJ is an employee of Otto Bock HealthCare GmbH. The authors declare that they have no competing interests.

## Authors’ contributions

NJ participated in the design and coordination of the study, participated in the data acquisition, data analysis, results interpretation, manuscript drafting, and manuscript revisions. JVN participated in the design of the study, recruitment and coordination of the subjects, participated in the data acquisition, data analysis, and manuscript revisions. SM participated in the design of the study, participated in the data acquisition, and manuscript revisions. DF participated in the design and coordination of the study, data analysis, results interpretation, and manuscript revisions. All authors read and approved the final manuscript.
